# Qualitative Outcomes of Colorectal Cancer Screening Outreach Using Patient Navigation to Follow-Up Colonoscopy in Rural Primary Care Practices

**DOI:** 10.3390/cancers17213590

**Published:** 2025-11-06

**Authors:** Emily Myers, Jennifer Coury, Maryan Carbuccia-Abbott, Amanda F Petrik, Robert Durr, Jamie H Thompson, Erin S Kenzie, Gloria D Coronado, Melinda M Davis

**Affiliations:** 1 Oregon Rural Practice-Based Research Network, Oregon Health & Science University, Portland, OR 97239, USA; coury@ohsu.edu (J.C.);; 2 Kaiser Permanente Center for Health Research, Portland, OR 97227, USAjamie.h.thompson@kpchr.org (J.H.T.); 3 School of Public Health, Oregon Health & Science University-Portland State University, Portland, OR 97201, USA; 4 Department of Epidemiology and Biostatistics, University of Arizona Cancer Center, Tucson, AZ 85719, USA; 5 Department of Family Medicine, Oregon Health & Science University, Portland, OR 97239, USA

**Keywords:** colorectal cancer screening, patient navigation, rural health, implementation science, primary care

## Abstract

**Simple Summary:**

Despite strong evidence supporting colorectal cancer (CRC) screening, follow-up colonoscopy rates after abnormal fecal immunochemical tests (FITs) remain low, particularly in underserved rural populations. This study evaluated the implementation of a patient navigation program within a large, pragmatic trial targeting Medicaid enrollees in rural primary care settings. A total of 35 patients were eligible for navigation due to abnormal FIT results (*n* = 26) or elevated CRC risk (*n* = 9); only 8 of 14 intervention clinics had any eligible patients. Among those with abnormal FITs, 50% received navigation, and 23% completed diagnostic colonoscopy. While higher-risk patients received navigation, none completed follow-up. Implementation was hindered by staffing disruptions, limited access to colonoscopy, patient mistrust, and data infrastructure challenges. Facilitators included cross-clinic collaboration and flexible adaptation of navigation protocols in low-volume environments. Future models may benefit from centralized coordination with endoscopy providers and payer-based quality improvement strategies.

**Abstract:**

**Background/Objectives:** Despite its effectiveness, colorectal cancer (CRC) screening rates are suboptimal in the United States. Navigating patients towards complete CRC screening can be effective in addressing barriers. However, to date, much research on patient navigation has occurred in urban settings or large health systems, thereby missing some populations that could benefit the most. **Methods:** We report on a patient navigation program delivered by clinic staff during a large pragmatic study to improve CRC screening in rural Medicaid populations. We use qualitative and implementation data from interviews, contract logs, and tracking systems to explore the context, barriers, and facilitators of patient navigation, as well as feasibility and acceptability for rural primary care clinic partners. **Results:** A total of 35 patients were eligible for navigation following an abnormal FIT (*n* = 26, 74%) or due to higher CRC risk (*n* = 9, 24%); only 8 of the 14 intervention clinics (57%) had any eligible patients. Of the 26 patients who needed navigation following an abnormal FIT, 13 patients (50%) received navigation, and 3 (23%) completed a colonoscopy; all 9 of the higher-risk patients received navigation, but none completed colonoscopy. Several barriers impacted adherence to the navigation protocol, such as staffing disruptions, limited colonoscopy availability, patient mistrust, and data tracking limitations. Our findings also highlight implementation facilitators, including protocol adaptations and cross-team collaborations for low-volume settings. **Conclusions:** Future models to increase patient navigation in rural settings could include more centralized system-level interventions that build on relationships between clinics and colonoscopy providers or payers and leverage quality improvement best practices.

## 1. Introduction

Colorectal cancer (CRC) is the second leading cause of cancer death in the United States, yet it is 90% curable with timely detection and appropriate treatment of precancerous growths [1,2,3,4]. Despite its effectiveness, CRC screening rates are suboptimal in the general population [5,6], with disproportionately high CRC incidence and mortality in certain populations [7,8,9].

Navigating patients to complete cancer screening can be effective in addressing screening barriers, especially in populations with disproportionately low screening uptake [10,11,12,13,14,15,16]. Patient navigation is an evidence-based approach designed “to promote access to timely diagnosis and treatment of diseases by eliminating barriers to care,” [17,18,19] and has been recommended for improving CRC screening rates by the Community Preventive Services Task Force [20]. Patients who receive navigation are more likely to complete a colonoscopy after an abnormal fecal test result [21,22,23]. Improving follow-up colonoscopy completion rates and shortening the time to receipt of colonoscopy can significantly improve health outcomes [10,24,25,26,27,28]. Navigators can impact these outcomes by supporting care coordination between primary and specialty care (e.g., gastroenterology or other colonoscopy providers), creating individualized care plans, sending reminders, scheduling appointments, and serving as a primary contact for patients needing CRC screening [29].

Implementation challenges, however, can prevent the widespread adoption of patient navigation programs. Prior research shows that the successful implementation of patient navigation relies on core components, such as partnerships, a defined protocol, clinical champions, and data tracking systems [30]. Much of the research to date has implemented centralized navigation programs in larger health systems [31], but small, rural practices might call for a different approach adapted to local contexts to support implementation [15,32]. While intervention fidelity is fundamental to ensuring the effectiveness of complex interventions, adherence to a complex protocol might become more challenging as interventions are tested in real-world practices, especially in rural settings [33].

The Screening More patients for CRC through Adapting and Refining Targeted Evidence-based Interventions in Rural settings (SMARTER CRC) study paired mailed fecal immunochemical tests (FITs) with patient navigation to follow-up colonoscopy to increase rates of CRC screening among Medicaid enrollees living in rural areas [34]. This large-scale, cluster-randomized trial to improve rates of CRC screening was conducted with three Medicaid health plans and 28 rural clinics. The SMARTER CRC intervention showed a 7.5% increase in screening compared to control clinics (full outcomes reported elsewhere) [35].

We previously reported in Coronado et al. [35] that the patient navigation component of SMARTER CRC was effective at 6 months; among the 34 enrollees, follow-up colonoscopy completion differed by 27.9 [95% CI = 1.2–54.6] percentage points at the 6-month follow-up (intervention, 43.3% vs. usual care, 15.4%; *p*  =  0.04), but did not significantly differ for the 12-month data. In this study, we use qualitative and implementation data from the SMARTER CRC study to explore the context of patient navigation during the clinical trial and the acceptability and feasibility of the navigation program for rural primary care clinic partners.

## 2. Methods

We report implementation outcomes using descriptive statistics, specifically the number of patients who were eligible for navigation, navigation delivery, and colonoscopy completion. We conducted a qualitative thematic analysis to evaluate the acceptability of patient navigation in rural primary care clinics [36]. We applied the updated Consolidated Framework for Implementation Research (CFIR) [37] to identify domains that influenced patient navigation implementation. We use the term “colonoscopy provider” to refer to primary care providers who perform colonoscopies and general surgeons who provide colonoscopies. This term aligns with that described in past research that explores colonoscopy provision to rural Medicaid patients [38]. SMARTER CRC obtained approval from the OHSU’s Institutional Review Board (protocol number: 20681) and was granted a waiver of informed consent.

### 2.1. Study Setting and Research Partners

The SMARTER CRC study was part of the National Cancer Institute-funded Accelerating Colorectal Cancer Screening and Follow-up through Implementation Science (ACCSIS) consortium [39]. SMARTER CRC was a partnership between the Oregon Rural Practice-based Research Network at Oregon Health & Science University (OHSU) and the Kaiser Permanente Center for Health Research. This study was conducted with the 14 rural clinics that implemented the SMARTER CRC intervention for their Medicaid population.

### 2.2. Patient Navigation Intervention

Patient navigation was delivered by clinic or health plan staff in the intervention clinics. Implementation of the navigation components was supported by multi-model virtual training delivered by research team members (JT, JR), detailed elsewhere [40]. After the initial training, practice facilitators supported navigation implementation through coaching and technical assistance [41]. Practice facilitators on the research team met with each participating clinic and customized the patient outreach in alignment with existing clinic workflows.

During the SMARTER CRC intervention, eligible patients were identified using health plan claims data and then verified as eligible to receive a mailed FIT by clinic staff (i.e., during a list “scrub” process) [35]. Patients with an abnormal FIT result were notified of the result through usual clinic procedures (i.e., their normal provider or an outreach coordinator), and then followed up using patient navigation. In addition to these patients, during the scrub process, some patients were directly referred to navigation by the clinic team if they were due for CRC screening but ineligible for mailed FITs because they were at higher risk of CRC. For those patients, the first navigation outreach contact would include a notification that the patient was due for CRC screening. Clinic staff were trained on how to enroll patients eligible for navigation during the virtual training.

The patient navigation intervention was adapted from the New Hampshire Colorectal Cancer Screening Program [30] based on prior work with rural clinics [23,42]. The adapted intervention was phone-based and covered four topics: (1) first call and barrier assessment, (2) bowel preparation review, (3) colonoscopy check-in, and (4) colonoscopy results review. Patient navigators were clinic staff who were trained by the research team; the health plan also offered trained navigators to supplement the clinical team. Patient navigators were trained to document all outreach efforts in a custom Patient Tracker created in a REDCap database [43,44].

### 2.3. Eligible Patients

**Figure 1:**
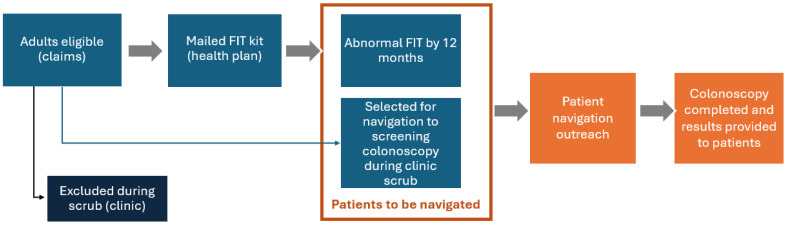
Patient navigation workflow.

In the SMARTER CRC pragmatic trial, patients were eligible if they were aged 50–75, due for CRC screening, and enrolled in Medicaid or dually eligible for Medicaid and Medicare. The eligible population of this study is the subset of SMARTER CRC eligible patients who should receive patient navigation because they are eligible for CRC screening after the scrub process, and either had an abnormal FIT result within 12 months or were marked by clinic staff to receive navigation to colonoscopy because they were ineligible for mailed FITs (see Figure 1) [45]. As clinical practices customized the SMARTER CRC intervention, only two clinics chose to use navigation for patients who needed screening colonoscopy and were not eligible for the mailed FITs. For consistency with the prior literature, we define follow-up colonoscopy as a colonoscopy following an abnormal stool test result and a screening colonoscopy as a colonoscopy performed on an asymptomatic person to test for the presence of colorectal cancer or colorectal polyps [46].

### 2.4. Data Collection and Outcome Measures

We collected qualitative data at multiple time points throughout this study (see Table 1). Qualitative interviews (i.e., Patient Navigation and Clinic Exit) were led by at least two qualitative analysts (AHO, EM, EK, NR). Clinic midpoint interviews were conducted by the research project manager and practice facilitators, using a workflow assessment guide. Interview guides were informed by two frameworks: the Framework for Reporting Adaptations and Modifications to Evidence-based Implementation Strategies (FRAME-IS) [47] and the Program Sustainability Assessment Tool [48]. Interviews were conducted virtually via Zoom 5.8.6, recorded with participant consent, professionally transcribed through Rev, and validated by qualitative team members for accuracy.

Navigation receipt was defined as an indication in REDCap that a clinic staff member successfully contacted the patient by phone. We reviewed and analyzed the REDCap data to record receipt of any navigation and colonoscopy completion. Research team and clinic staff members conducted chart reviews to determine all FIT results and confirm colonoscopy completion.

### 2.5. Qualitative Analysis

Qualitative data analysis was completed by at least 2 trained team members (JSR, JS, AHO, EM, EK, NR). Data cleaning and thematic analyses were conducted concurrently with data collection. Specifically, qualitative team members created coding dictionaries using a hybrid approach (i.e., inductive and deductive) and coded all transcripts using ATLAS.ti (24) and NVivo (12.0). While inter-coder reliability was not quantitatively assessed, qualitative team members co-coded interviews and discussed coding decisions to ensure that coding strategies aligned.

A constant comparative analysis approach with open-coding techniques and iterative reviews of coded data was used to generate qualitative findings (i.e., summary of themes with illustrative quotes) [49]. Qualitative team members reviewed and discussed thematic summaries with the study team to ensure accuracy of interpretation.

We conducted 5 patient navigation interviews, 11 midpoint interviews, and 12 exit interviews with all year-one intervention clinics (*n* = 15). Descriptive quantitative statistics informed our analysis sample, and we only included clinics with patients to navigate in the qualitative analysis. One qualitative team member (EM) created an analytic matrix to triangulate the findings from all three data sources, applied the CFIR constructs [37], and generated implementation themes. For CFIR constructs, we utilized the outer and inner setting domains to center the experience of participating Medicaid health plans and clinics. Within the outer setting domain, three constructs were central to our findings (critical incidents, local attitudes, and partnerships and connections), and four constructs did not apply to this study. Within the inner setting domain, seven constructs appeared most applicable (informational technology (IT) infrastructure, work infrastructure, relational connections, compatibility, relative priority, available resources, and funding). Emerging themes were discussed with the principal investigator, project manager, and two qualitative team members (EK, AHO) to inform further analysis and reconciliation. Findings were ultimately distilled to three pertinent inner setting constructs (IT infrastructure, relational connections, and compatibility), and four constructs did not apply to our study.

## 3. Results

Across all SMARTER CRC intervention clinics, a total of 35 patients were eligible for navigation. Of the patients eligible for navigation, 26 patients (74%) were eligible for navigation due to an abnormal FIT result within 12 months, and 9 (26%) were referred for a screening colonoscopy (see Table 2). Only 8 of the 14 intervention clinics (57%) had any patients eligible for patient navigation, and the qualitative findings below are from those clinics. Of the 26 patients who needed navigation due to an abnormal FIT within 12 months, 13 patients (50%) received navigation and 3 (23%) completed a colonoscopy within 12 months.

The qualitative analysis identified three outer setting (e.g., critical incidents, local attitudes, and partnerships and connections) and three inner setting domains (e.g., compatibility, relational connections, and IT infrastructure) that were most salient to program implementation and adaptations (see Table 3).

### 3.1. Outer Setting Themes

**Critical Incidents—unanticipated disruptions:** The COVID-19 pandemic was a large-scale event that impacted implementation in our trial [50]. Largely due to the pandemic, clinics commonly reported system-level barriers such as clinic staff turnover and shortages, limited availability for colonoscopies, and extended referral waitlists. These critical incidents were primary barriers to adhering to patient navigation implementation. Staff turnover and shortages resulted in inconsistent outreach and scheduling with patients, gaps in REDCap documentation, and the need for rehiring and retraining patient navigators.

**Local Attitudes—patient-level barriers:** Patient navigators described both positive and negative patient attitudes toward being navigated and completing a follow-up colonoscopy. Navigators reported the following barriers: competing health issues; mistrust of the health plan and mailing vendor information; and hesitation due to bowel preparation.

**Partnerships and Connections—health plan partnerships:** The intervention was designed to be collaborative with the health plans. Despite offering and training one health plan-level navigator, clinics did not specifically utilize the health plan-level patient navigation support. However, some clinics did engage with their health plan for general and local resources, as illustrated in the following quote:

We were able to identify working with [health plan] that, when you go to do a prior authorization for a colonoscopy after a FIT, sometimes it wouldn’t [create a] prior authorization … And so, they have removed that. And we have a pretty good relationship with [health plan], and so I could just call them and say, “Hey, I think this might be happening. Can we work around this?” So, the opportunity to sit down and problem solve some of these things has been huge in the process mapping.

—Navigator 2

Patient navigators also expressed that ideally, health plan-level navigators would support clinics with patient outreach, share medical knowledge, and help clinics meet incentivized metrics.

### 3.2. Inner Setting Themes

**Compatibility—clinic characteristics:** We found that the protocol was well received and better utilized within clinics that had no staffing disruptions and utilized several staff members to ensure patients completed a follow-up colonoscopy. One clinic with an existing and well-established infrastructure reflected the following:

I feel like our experience isn’t fair to other clinics because we have so many different departments. We have a referrals department. We have care managers. We have the docs who do the colonoscopies on site. We have a front desk staff. Everything is pretty much already in place, so we don’t have to do a lot. We weren’t having to build a whole new system to be part of this project.

—Navigator 5

On the other hand, clinics that experienced staffing disruptions, replaced and retrained patient navigators, or did not realize the value of the protocol struggled to implement patient navigation. Clinics with these characteristics expressed the following:

We were so shorthanded, there were MAs taking more than one provider, rooming for more than one provider at a time. So there was just no choice to where they just couldn’t do it [navigate patients] last year.

—Navigator 4

Additionally, the quality of workflows, systems, and processes that clinics had with their providers was also a key factor when implementing patient navigation. Some clinics expressed that their providers were unaware of the program and did not know patients were receiving FITs. Therefore, clinics experienced misalignment and a lack of support when implementing patient navigation.

**Relational Connections—protocol adaptations and collaboration with colonoscopy providers:** Clinics commonly reported that the patient navigation protocol would be repetitive or overstep colonoscopy provider responsibilities. Therefore, clinics implemented the patient navigation protocol to varying degrees, depending on their existing workflows. One clinic opted to discuss only barrier assessment and bowel preparation with patients, with the expectation that colonoscopy providers would complete colonoscopy check-in and results. Navigators at another clinic felt that it was only necessary to make the initial navigation contact and assumed colonoscopy providers would be responsible for the remaining navigation topics. Similarly, navigators at another clinic reported that they opted not to implement the protocol because they believed that colonoscopy providers were performing sufficient outreach. Therefore, these clinics felt there was no need for additional follow-up, and patients with an abnormal FIT often went unnoticed. One clinic specifically shared the following:

Once we do the referral and [colonoscopy providers] get involved, we step out of the equation and they take over, from getting that initial appointment to talking to them about what the procedure’s going to entail, what to expect. We did not catch that this had not been addressed by [colonoscopy providers], so I’m working on a new process to make sure that there is a more active involvement on our end and follow up with the patient and with [colonoscopy providers] to make sure that this is going through. We had a missed opportunity, the patient was in the clinic, and because there had not been [patient navigation follow-up], I put a note on the appointment that we had a missed opportunity for a face-to-face discussion from the provider to the patient, and that hurt, that hit hard.

—Navigator 1

Ultimately, not all clinic staff understood the impact they could have in closing the gap between placing colonoscopy referrals and patients completing the procedure. This showed up when clinics would place the referral and not follow the patient navigation protocol, or when patients declined the follow-up colonoscopy or navigation support, and the clinic did not continue to reach out to or motivate the patient.

I know that the beginning step would be that it’s ordered and we always hit those. But then, the second and third before the completion were hard to follow up with sometimes because they were related to communicating with the patient and stuff from the person that’s doing the colonoscopy so it wasn’t ever really related to us. That part was hard.

—Navigator 3

Dr. [Last Name] always says, we can’t try harder than the patient, and if the patient’s not really interested, you know, we can try as hard as we want and it’s not really probably going to make much difference.

—Navigator 7

Despite varying levels of clinic adoption, patient navigators were able to help some patients complete a follow-up colonoscopy and shared the following implementation stories:

I remember one patient in particular who tested [abnormal] and then had the colonoscopy and had polyps removed and was just super thankful that we had done that because he had recently had some family members that were having cancer issues and stuff. I know he was just really thankful, and his went very smoothly.

—Navigator 3

This is a tough patient, <laugh>, they did not want to do anything, they’re going to be fine, fine, fine. And at some point, you have to say, okay, you really need it. And you know, the fifth call, they’re not going to get it. That’s where I thought where we were at with this patient. But the last time I looked at their chart, they had made an appointment with the referral office. So, we referred them. Now he’s made an appointment. I think that’s a huge step for that patient because not only is he starting to come around, but he’s going to see that [colonoscopy provider] and they’re going to give him some information.

—Navigator 4

**IT Infrastructure—patient navigation data systems:** The REDCap system used throughout implementation guided clinics through the patient navigation protocol. However, clinics found it to be overwhelming and redundant to track patient encounters in both REDCap and their electronic health record (EHR) system. Some clinics used their EHRs; however, the clinic EHRs were typically not adequate, and most clinics had to create an Excel spreadsheet to track patients enrolled in navigation.

Clinics shared mixed responses when asked if they plan to incorporate navigation in the future. Some clinics plan to continue implementing some or all components of the patient navigation protocol. A couple of clinics were uncertain whether they could continue due to limited staffing and low capacity, and one clinic was still hesitant about the need for patient navigation at the clinic level.

## 4. Discussion

We report on a patient navigation program delivered by clinic staff as part of a large pragmatic study to improve CRC screening in rural Medicaid populations. Our analysis of qualitative and implementation data explored the context, barriers, and facilitators of implementing patient navigation by staff in rural primary care clinics. We present these data with the view that intervention adaptation is often necessary to address context [33].

Notably, these rural clinics had very few patients who needed a follow-up colonoscopy after an FIT mailing—only 26 out the large initial pragmatic trial population. In clinics that opted to perform FIT mailing, some additional people (*n* = 9) were selected by clinics for navigation to a screening colonoscopy. Therefore, the navigation protocol was not utilized by all year-one intervention clinics, and clinic navigators had few chances to practice the navigation skills they learned in training.

Of the patients who needed navigation, only 53% of patients were contacted, a substantial drop-off in the reach of the navigation intervention. On the other hand, no patients in the higher-risk group completed a colonoscopy within 12 months even though they all did receive navigation. A number of factors could have led to these lower outreach and completion rates: The low number of patients requiring navigation likely made it harder to implement patient navigation consistently. Navigators had only one or two people in a year requiring navigation, which made it challenging for the navigators to have enough practice with the skills to confidently address barriers to colonoscopy. We also found that the patient navigation protocol was more feasible in clinics with stable and adequate staffing, which supports prior research about facilitators of cancer screening in primary care practices [11,13,51,52,53]. Prior research within rural practices has reported substantial barriers to providing screening services and follow-up care, including competing demands, shortages of medical staff, and a lack of funding for follow-up care [54,55]. Other barriers to colonoscopy completion were limited availability for colonoscopies and extended referral waitlists.

Navigators also reported patient-level barriers, including hesitation about the bowel preparation, competing health issues, and mistrust of the health plan (which was involved in the FIT mailing). These findings align with the commonly known and often-cited barriers to completing colonoscopies (e.g., stigma, hesitancy, and a shortage of specialists) [56,57,58]. Moreover, a large share of the participants in the main pragmatic trial (81%) had no prior history of CRC screening [35], which is known to lower the response to screening completion efforts. This population often requires additional outreach to complete CRC screening, and patient navigation is only one of many evidence-based interventions recommended by the Community Preventive Services Task Force [59].

The disruptions shared by participants highlight how unstable and overburdened rural primary care workforces make it difficult for clinics to maintain clinical staff navigators. This barrier might be alleviated by designing a system-level intervention using the in-depth knowledge of the referral network and building relationships between primary care clinics and the colonoscopy providers (either gastroenterology or general surgeons) to better distribute the work among staff [10,53,60,61,62]. Navigation training focuses on several different preventive services, and motivational interviewing training could address gaps as well. Centralized staff could be trained in regional resources and barriers. These findings suggest that adapting the intervention towards centralized services could be more resource-efficient [13]. This suggestion is also supported by the finding that the navigation protocol was better utilized within clinics that had well-established and centralized infrastructure. The question of capacity could be addressed by a centralized approach [13,63] or using staff at the health plan level [13]. Centralized support will need to be designed in collaboration with rural providers to reflect community priorities and relationships [64,65,66,67].

Despite research showing the effectiveness of patient navigation [19,68,69], some clinics lacked the buy-in required for successful implementation. Patient navigation is distinct from the typical patient outreach that occurs within primary care [31,41]. Along with this, clinic-level navigators were instructed to communicate and collaborate with their providers to ensure patients completed follow-up colonoscopies. Additional training is likely needed for staff at all levels (managers to navigators) to understand the value of navigation and how this differs from specialists’ education. Multiple factors can affect patient risk levels for abnormal colonoscopy findings and polyp recurrence, which could impact screening recommendations [70,71,72]. Provider recommendations can motivate patients and support navigator messaging regarding the urgency of follow-up colonoscopies.

This study has a few important limitations. We found that patient navigators did not regularly and adequately use the REDCap Patient Tracker system. As navigation attempts were inconsistently recorded in the REDCap system, we do not present total navigation attempts, only the successful delivery of at least some navigation. Our learning from this is that tracking systems need to be part of the regular clinic workflow (i.e., EHR systems), and systems need to integrate better with EHRs while connecting patients to follow-up care. This supports prior research on the complexity of CRC screening data [73] and research on EHR support for clinic QI efforts [74]. Our small sample was also a limitation, as we had a small number of navigators and patients to be navigated. However, the numbers are a realistic representation of patient navigation in rural and frontier areas and are similar to the number of patients in prior research studies in rural areas [22]. As the numbers are small, we chose a mixed-methods evaluation, and the qualitative findings provide additional insights for this limited sample. In addition, as our study had a waiver of written informed consent [41], participants might be more representative of the regular patient population with a less chance of selection bias than many prior trials of patient navigation. Finally, we do not have the data to report specific clinic plans to continue patient navigation; however, a SMARTER CRC second-year analysis is planned that will be able to further explore the sustainability of patient navigation in participating clinics.

Future research could continue to explore the feasibility and acceptability of centralizing patient navigation at the health system or health plan level [13,22], with a direct comparison of centralized versus clinic-level patient navigation support. Ultimately, more research is needed to create a model that is successful and supportive of patient navigation in rural areas. It is also essential for future programs to acknowledge the challenges within the rural primary care workforce to ensure that future patient navigation programs can be sustained [75]. Models that leverage technology, such as EHR patient portals or texting, to reach patients before outreach by patient navigators could extend the reach of follow-up colonoscopy reminder programs.

Beyond logistical implementation, we found a disconnect between the primary care clinic and colonoscopy provider responsibilities regarding CRC screening outreach. Future studies could explore the workflow between primary care clinics and colonoscopy facilities to create a team-based system that enables patients to seamlessly complete a follow-up colonoscopy [76]. In low-volume settings, the patient navigation model could train clinic staff in barrier resolution and results tracking and train colonoscopy provider staff in scheduling and patient communications.

## 5. Conclusions

Our research on the implementation of patient navigation to colonoscopy in rural primary care practices uncovered barriers to navigation adherence, including staffing disruptions, the limited availability of colonoscopies, patient mistrust, and EHR limitations. It also highlighted helpful implementation facilitators, including protocol adaptations and collaborations for low-volume settings. These findings expand the knowledge base on the implementation strategies of patient navigation in rural clinical practices beyond prior research, thereby including some populations that could benefit most from this approach.

## Figures and Tables

**Table 1:** Qualitative data sources.

Data Source	Participants	Data Collection Time Point	Purpose
Navigation Tracker (REDCap forms)	Patient Navigators	Throughout program Year 1 (2021–2022)	Track and record navigation activities and colonoscopy outcomes (e.g., receipt of colonoscopy), including attempted and completed calls
Patient Navigation Interviews	Patient navigators with patients eligible for navigation	End of program Year 1 (April–July 2022)	Understand clinic experiences with patient navigation, assess clinic-level barriers and facilitators, evaluate patient navigation training, and evaluate the sustainability of the protocol
Clinic Midpoint Interviews	Clinic leads from intervention and control clinics	End of program Year 1 (March–April 2022)	Assess clinic workflows, disruptions (e.g., COVID-19 pandemic and staff challenges), and program impressions
Clinic Exit Interviews	Clinic leads from intervention and control clinics	End of program Year 2 (May–July 2023)	Explore program impressions, adaptations, and the sustainability of mailed FITs and patient navigation

**Table 2:** Year 1 patient navigation outcomes for intervention clinics.

Outcomes	Patients Eligible for Navigation ^a^	Patients Who Received Navigation ^b^	Colonoscopy Completed ^c^
Abnormal FIT results	26	13 (50%)	3 (23%)
Screening colonoscopy	9	9 (100%)	0 (0%)
Total	35	22	3

^a^: Patients with abnormal FIT results within 12 months were considered eligible for navigation. ^b^: Patient who received navigation % = *n* patients eligible for navigation/*n* patients who received navigation. ^c^: Follow-up colonoscopy completed % = *n* enrollees who completed a follow-up colonoscopy within 12 months of abnormal FT result/*n* enrollees who received the patient navigation.

**Table 3:** Qualitative findings.

**CFIR Construct:** **Key Theme**	**Key Findings**
Outer Setting	
Critical Incidents: Unanticipated disruptions	The COVID-19 pandemic affected the implementation of our trial, leading to system-level barriers, including staff turnover, shortages, and limited colonoscopy availability. These challenges led to inconsistent patient outreach, documentation gaps, and referral delays, ultimately hindering adherence to implementation.
Local Attitudes: Patient-level barriers	Patient navigators described both positive and negative patient attitudes toward being navigated and completing a follow-up colonoscopy. Navigators reported the following patient-level barriers: competing health issues; mistrust of the health plan and mailing vendor information; and hesitation due to bowel preparation.
Partnership & Connection: Health plan partnerships	Despite offering and training one health plan-level navigator, clinics did not specifically utilize the health plan-level patient navigation support. However, some clinics did engage with their health plan for general and local resources. Patient navigators also expressed that ideally, health plan-level navigators would support clinics with patient outreach, share medical knowledge, and help clinics meet incentivized metrics.
Inner Setting	
Compatibility: Clinic characteristics	The protocol was well-received and better utilized within clinics that had no staffing disruptions and utilized several staff members to ensure patients completed a follow-up colonoscopy. Clinics that experienced staffing disruptions, replaced and retrained patient navigators, or did not realize the value of the protocol struggled to implement patient navigation. The quality of workflows, systems, and processes clinics had with their providers was also a key factor when implementing patient navigation.
Relational Connections: Protocol Adaptations & Collaboration with Colonoscopy Providers	Clinics commonly reported that the patient navigation protocol would be repetitive or overstep colonoscopy provider responsibilities. Therefore, clinics implemented the patient navigation protocol to varying degrees, dependent on their existing workflows. Not all clinic staff understood the impact they could have in closing the gap between placing colonoscopy referrals and patients completing the procedure.
IT Infrastructure: Patient navigation data systems	The REDCap system used throughout implementation guided clinics through the patient navigation protocol. However, clinics found it to be overwhelming and redundant to track patient encounters in both REDCap and their electronic health record (EHR) system. In clinics that used their EHRs, the EHRs were typically not adequate, which required creation of a separate spreadsheet to track patient navigation.

## Data Availability

The raw data supporting the conclusions of this article will be made available by the authors on request.
